# When a “Low T” Diagnosis Can Be the Clue to a More Complex Problem

**DOI:** 10.7759/cureus.51215

**Published:** 2023-12-28

**Authors:** Adrian G Dumitrascu, Ana-Maria Chindris, Claudiu Matei, Razvan M Chirila

**Affiliations:** 1 Hospital Medicine, Mayo Clinic, Jacksonville, USA; 2 Endocrinology, Diabetes and Metabolism, Mayo Clinic, Jacksonville, USA; 3 Neurological Surgery, Lucian Blaga University, Sibiu, ROU; 4 Internal Medicine, Mayo Clinic, Jacksonville, USA

**Keywords:** testosterone replacement, hypopituitarism, partial empty sella turcica, male hypogonadotropic hypogonadism, low t

## Abstract

Male hypogonadism remains a poorly evaluated and managed clinical condition despite the availability of clinical guidelines. We present a case of a male patient diagnosed with secondary hypogonadism related to partial empty sella syndrome, whose clinical course was complicated by a hypotensive near-syncopal event. Although initial hypopituitarism symptoms could be subtle and nonspecific and could involve only one hormonal axis, a thorough evaluation of the pituitary function may identify additional deficiencies such as a subclinical chronic adrenal insufficiency that may become manifest during situations of increased physiological stress with potential life-threatening consequences.

## Introduction

Nonspecific symptoms like fatigue, low energy, sleep disturbance, and impaired concentration are complaints commonly encountered in primary care practices, in both female and male patients. The differential diagnosis is broad and includes psychiatric, neurologic, metabolic, hormonal, and hematological causes, sometimes requiring extensive investigations to reach a definitive diagnosis. But when such symptoms are associated with decreased libido in males, and sometimes with decreased sexual activity, a concern for male hypogonadism or “testosterone deficiency” is raised and testosterone level is checked by the patients' primary care providers. Following a low serum testosterone level, testosterone replacement therapy is usually offered for testosterone deficiency, or “low T" as it was frequently called in direct-to-consumer product advertisement [1]. Following such advertisement campaigns and guideline-discordant prescription practices, the use of testosterone replacement therapy has increased dramatically over the past 2 decades in the United States [2]. However, a low testosterone level, confirmed in at least two instances, should always prompt additional evaluation to further differentiate between a primary or secondary hormonal dysfunction. A secondary hypogonadotropic hypogonadism related to a hypothalamic or a pituitary cause can be missed if a low testosterone level is not followed by an evaluation for gonadotropin deficiency. Impaired gonadotropins production is frequently associated with other pituitary hormones' hyposecretion. Adrenocorticotropic hormone (ACTH), thyroid-stimulating hormone (TSH), and growth hormone (GH) deficiencies can be subtle, without obvious clinical symptoms. Unfortunately, a complete evaluation of male hypogonadism is frequently not done before testosterone replacement therapy is prescribed [3]. We present a case of a male patient diagnosed with hypogonadism that was not appropriately evaluated, and whose clinical course was complicated by a near-syncopal event. Upon additional evaluation, the patient was diagnosed with a pituitary condition responsible for multi-hormonal deficiencies. This case emphasizes the need for a thorough and complete evaluation of patients presenting with clinical and biochemical confirmed hypogonadism, in order to avoid missing a broader pituitary dysfunction that, if left untreated, could expose patients to serious risks.

## Case presentation

A 56-year-old male presented to the Internal Medicine Clinic as a new patient for an annual physical examination. His past medical history was significant for hyperlipidemia (treated with simvastatin 20 mg daily) and mild chronic asymptomatic hyponatremia (untreated) with serum sodium in “the low 130s.” He was recently seen and discharged from a hospital emergency room (ER) after a pre-syncopal event. His blood pressure at that time was within normal limits without orthostatic changes and he had only mild bradycardia on telemetry. The exercise stress test done during the admission revealed a hypotensive blood pressure response to exercise and it was followed by a cardiology consultation with a recommendation for cardiac imaging. CT coronary angiogram showed no plaques or stenosis and had a coronary artery calcium score of zero, ruling out an ischemic cardiac cause for his pre-syncope presentation. He received intravenous hydration, his blood pressure remained stable, and he was discharged home with recommendations to follow up with his primary care physician for additional evaluation of this condition. In retrospect, during the interview, the patient mentioned that this ER visit followed after a stressful, work-related event, a prolonged flight back home coupled with a decreased oral fluid intake during his travel. 

Otherwise, the patient felt well overall, exercised regularly, and got good sleep. He had a normal diet with no over-the-counter supplements, no restrictions on proteins or sodium, and no consumption of alcohol. He worked as an executive and traveled frequently. He was married and he had fathered 2 children. Regarding his fatigability, his previous primary care doctor diagnosed him with low testosterone levels. The patient did not remember being told about or being investigated for a particular cause of low testosterone. He was prescribed topical testosterone but did not start this medication. He added that he did not feel the need for the prescription as he had been exercising and had not noted any difficulties with building muscle mass. When questioned about sexual activity and libido, the patient noted that his libido was decreased, and he had not had any sexual activity of any kind for the past 2-3 years.

The patient’s vitals revealed a temperature of 36.7°C, blood pressure of 130/78 mmHg, and heart rate of 52 beats/min. He was 176.8 cm tall and weighed 82 kg resulting in a BMI of 26.23 kg/m^2^. The patient did not have orthostatic hypotension. Physical examination showed a well-developed man with normal body habitus, who was alert and oriented. Head and neck examinations were within normal limits. A cardiac examination was significant for bradycardia with regular rhythm and normal cardiac sounds. Pulmonary effort and chest excursion were normal and breath sounds were clear bilaterally. An abdominal exam showed no hepatomegaly, with no central obesity and it was normal otherwise. The patient declined the genital examination. He had a normal musculoskeletal and skin exam, without alopecia and a normal presence of body hair. The neurologic examination was normal, and the patient had a normal mood and affect.

Upon review of the patient's old records, we noted that his serum sodium was checked 3 times in the previous 6 months with values ranging between 128-132 mmol/L (reference range: 135-145 mmol/L).

The AM testosterone levels were repeated twice, and the recent emergency department (ED) obtained labs are listed in Table 1. Liver function tests were within normal limits.

**Table 1:** Initial laboratory data

Parameter	Patient value	Reference range
AM total testosterone	130 ng/dl	240-950 ng/dl
Repeated AM total testosterone	202 ng/dl	240-950 ng/dl
AM free serum testosterone	2.45 pg/mL	4.6-22.4 ng/dl
Repeated AM free serum testosterone	5.25 ng/dl	4.6-22.4 ng/dl
Hemoglobin	12.6 g/dL	13.2-16.6 g/dL
White blood cell count	5.2 x 10^9^/L	3.4-9.6 x10^9^/L
Platelet count	234 ×10^9^/L	157-371×10^9^/L
Total cholesterol	201 mg/dl	<200 mg/dl
Triglycerides	238 mg/dl	<150 mg/dl
Serum sodium	129 mmol/L	135-145 mmol/L
Serum potassium	3.8 mmol/l	3.5-5.2 mmol/L
Serum creatinine	1.1 mg/dL	0.9-1.04 mg/dL
Urine osmolality	520 mOsm/kg	150-1150 mOsm/kg
Urine sodium	99 mmol/L	No reference range
HbA1c (glycosylated hemoglobin)	5.6%	4.0-5.6%

**Figure 1:**
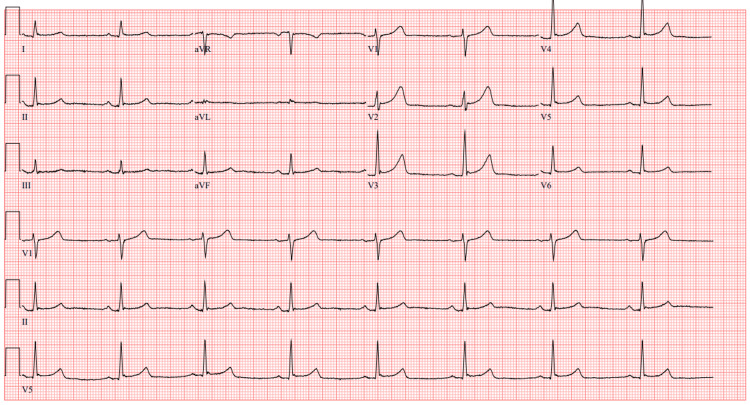
ECG on presentation to the emergency department; sinus bradycardia with heart rate 48, otherwise normal ECG

An ECG showed mild sinus bradycardia with no other abnormalities (Figure 1).

Considering the above symptoms and laboratory findings with persistently low testosterone levels, his presentation raised concerns for a diagnosis of hypogonadism. The presence of chronically low sodium in a euvolemic patient coupled with high urine osmolality and urine sodium pointed to a differential diagnosis of a syndrome of inappropriate antidiuretic hormone secretion (SIADH) or hormonal deficiency with hypothyroidism or adrenal insufficiency. Additional laboratory testing was ordered to evaluate his hypothalamic-pituitary-gonadal axis, the thyroid, and adrenal hormones. These are listed in Table 2. 

**Table 2:** Pituitary hormones

Parameter	Patient value	Reference range
Luteinizing hormone (LH)	2.0 IU/L	1.3-9.6 IU/L
Follicle-stimulating hormone (FSH)	2.9 IU/L	1.2-15.8 IU/L
Thyroid-stimulating hormone (TSH)	4.2 mIU/L	0.3-4.2 mIU/L
Thyroxine hormone (Free T4)	0.5ng/dL	0.9-1.7 ng/dL
Thyroperoxidase antibody	1.4 IU/mL	<9.0 IU/mL
Morning cortisol level	1.4 mcg/dL	7-25 mcg/dL
Morning adrenocorticotropic hormone (ACTH) level	24 pg/mL	7.2-63 pg/mL
Prolactin (PRL)	11.9 ng/mL	4-15.2 ng/mL
Insulin-like growth factor 1 (IGF1)	41 ng/mL	68-247 ng/mL
Growth hormone (GH)	0.1 ng/ml	0-10 ng/mL

Based on the inappropriately low-normal luteinizing hormone (LH) and follicle-stimulating hormone (FSH) levels in a patient with low testosterone, we diagnosed the patient with secondary/central hypogonadism. Furthermore, the very low morning cortisol level coupled with low normal ACTH, was consistent with a diagnosis of secondary adrenal insufficiency. The patient’s chronic mild hyponatremia was attributed to a chronic adrenal insufficiency diagnosis. His recent ED presentation with near-syncope and hypotension related to a stressful event was caused by a transient hypotensive episode in the patient with subclinical chronic adrenal insufficiency. His low free T4 was associated with an inappropriately low TSH explained by a decreased secretion of pituitary TSH. With low IgF1/low GH levels patient had a third hormonal axis affected. This constellation of hormonal deficiencies completed the diagnosis of panhypopituitarism.

Additional historical information was obtained from the patient attempting to differentiate the causes of hypopituitarism. He denied a family history of chronic liver disease or iron overload, heritable familial conditions, personal use of anabolic steroids or glucocorticoids recently, opioid pain medications, a history of, or symptoms of sleep apnea, any recent head trauma, or any brain surgeries or brain radiation. The patient was up to date with his cancer screening.

**Figure 2:**
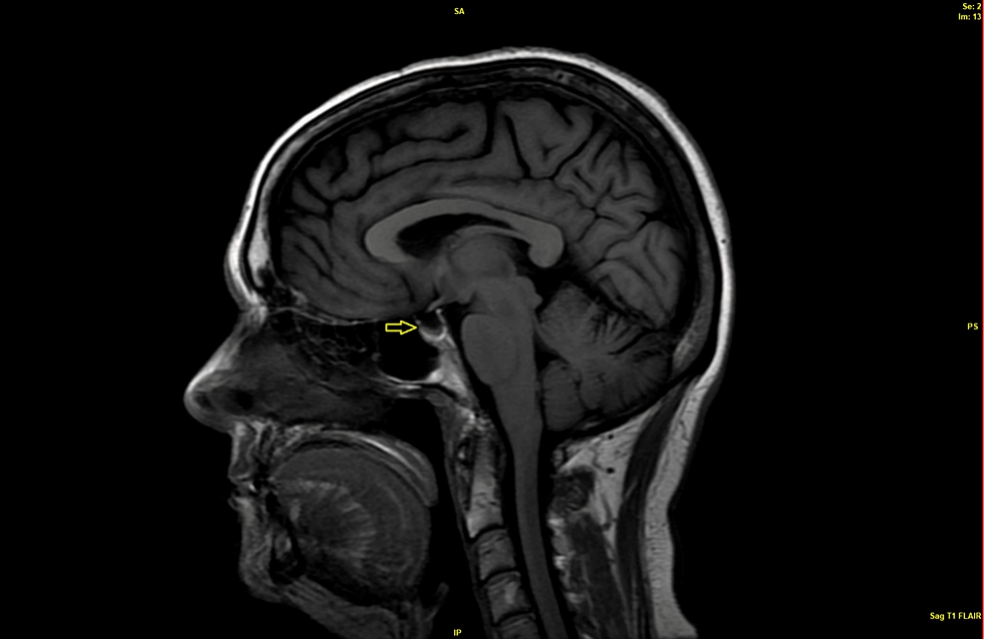
Brain MRI, sagittal view with partial empty sella

A magnetic resonance imaging of the brain (MRI) without and with contrast and with close sections for pituitary sella was done. The MRI ruled out any signs of increased intracranial pressure, any hypothalamic or pituitary infiltrative lesions, masses, hemorrhages, or infarcts. The patient had a partially empty sella with compression of his pituitary gland against the sellar cavity wall as depicted in Figure 2. 

A partial empty sella syndrome associated with hypopituitarism was diagnosed and the patient was first started on therapy with hydrocortisone, initially at a higher dose that was subsequently tapered to the “physiological” maintenance dose. His constitutional symptoms and hyponatremia improved within weeks. He was referred to Endocrinology to discuss the risks and benefits of testosterone supplementation and for additional follow-up for his hypopituitarism. The patient was counseled regarding the risks associated with adrenal insufficiency, about emergent glucocorticoid supplementation during periods of higher stress, and about wearing a medical alert bracelet.

## Discussion

This case emphasizes several commonly encountered pitfalls in the diagnosis and management of hypogonadism and brings awareness to the diagnosis of empty sella syndrome. Although previously considered to be only an incidental finding on CT or MRI of the brain of questionable clinical significance, hormonal dysfunctions associated with this condition can result in subtle to even severe clinical manifestations.

Male hypogonadism is due to decreased levels of circulating androgen hormones from either a primary testicular dysfunction or secondary from a hypothalamic or pituitary dysfunction with decreased secretion of gonadotropic hormones. Both types are sub-classified into organic and functional hypogonadism.

Male hypogonadism can present with nonspecific symptoms: reduced energy, impaired cognition/concentration, depressive mood, loss of muscle mass, and increased abdominal fat. Symptoms of erectile dysfunction, decreased libido, decreased spontaneous erection and gynecomastia are more suggestive of the diagnosis [4]. These symptoms associated with strong, unequivocal low testosterone levels (repeated low AM testosterone levels) will diagnose hypogonadism [4]. For patients having conditions known to alter the sex hormone binding globulin levels, free testosterone should also be measured. When evaluating testosterone deficiency, functional hypogonadism causes (hyperprolactinemia, alcohol, and marijuana abuse, glucocorticoids, opioids, anabolic steroid use, severe obesity and obstructive sleep apnea, chronic systemic illnesses, or severe nutritional deficiencies/excessive exercise) should be ruled out first [4]. LH and FSH levels should be measured to differentiate between primary or secondary hypogonadism. Low or low normal levels of LH and FSH in a patient with low testosterone levels should prompt a further evaluation for hypothalamic or pituitary causes. About 85% of cases of hypogonadism are classified as secondary hypogonadism [5]. Unfortunately, these last steps in the evaluation of testosterone deficiency were usually omitted by providers (in some studies in up to 88% of the patients!) potentially missing important associated diagnoses [6,7]. It is critical that this evaluation is completed to allow for an accurate assessment of the pituitary-gonadal axis [2].

More concerning is that testosterone replacement therapy is offered to patients for diagnoses other than hypogonadism and after incomplete evaluation. Some studies quote that only a limited number (4-18%) of patients had a confirmatory second testosterone test as per guidelines, and that other patients received testosterone supplementation despite a testosterone level within the normal range [8-11]. 

The pattern of testosterone prescription in the US is different and more aggressive when compared with other parts of the world [12]. Testosterone prescription has increased dramatically from 2000 until 2013 and in 2009 the administration form has changed from topical applications to injectable, long-acting formulations [2]. A direct-to-consumer and direct-to-provider marketing campaigns were responsible for this prescription increase [13]. After a prescribing peak in 2013, the use of testosterone has declined, likely following several published articles and FDA communication warning about increased cardiovascular risk in patients on testosterone supplementation therapy [2,14]. Both the Endocrine Society and the American Urological Association released guidelines in 2018 addressing the evaluation and management of patients with hypogonadism [4,15]. Following this transient decline, and after the guidelines were issued, there was again a significant increase in testosterone prescribing, with family practice, internal medicine, urology, and endocrinology providers writing most of the prescriptions (listed from the higher to the lower number of prescribers) [16]. There was a significant uptake in prescriptions by advanced care providers: nurse practitioners and physician assistants [16]. The recent telemedicine and direct-to-consumer online platforms have expanded access to health for males with sexual dysfunction and hypogonadism. There are reports that these platforms, primarily staffed by nurse practitioners and physician assistants, are also employing guideline-discordant prescription practices [17]. 

Once a diagnosis of secondary hypogonadism is made, a contrast-enhanced MRI of the brain will further help differentiate the central etiology. Among the radiographic findings associated with hypopituitarism (pituitary masses, infiltrative pituitary or hypothalamic disease, intracranial bleeding, extrapituitary masses causing brain compression) primary empty sella has an incidence of 8-35% [18]. This radiological finding refers to the presence of complete or partial filling or the sella turcica of the sphenoid bone with CSF with concomitant compression of pituitary tissue against an osseous wall. An empty sella can be a primary (without an immediately obvious cause, or secondary) sequela of another intracranial condition (post-surgery, radiation therapy, trauma, spontaneous necrosis, infection, autoimmune disease). Empty sella associated with hormonal deficiencies or neurological symptoms is defined as empty sella syndrome. Previous reports listed hypopituitarism in about 20% of patients [19] with primary empty sella (PES), but a recent review series noted hormonal deficiencies in 52% of the patients, and in 30% of patients multiple hormonal deficiencies were concomitantly present [20]. Growth hormone deficiency, hypogonadism, hypoadrenalism, and hypothyroidism frequency vary depending on the study [18,20,21]. Both Auer et al. and Carosi et al. concluded that due to the high frequency of hormonal abnormalities, all patients with incidental primary empty sella diagnosis should have an endocrinological evaluation at diagnosis [20,21].

Recently, numerous case reports have described refractory hypotension and unexplained severe hyponatremia associated with sudden exacerbation/crisis of secondary adrenal insufficiency during periods of stress. All of them occurred in patients with empty sella syndrome and undiagnosed chronic adrenal insufficiency [22-26].

## Conclusions

Despite the availability of clinical guidelines, male hypogonadism evaluation and management is highly variable among providers, with some specialists (endocrinologists and urologists) following guidelines more frequently than primary care providers. If a complete workup for hypogonadism is not done, there are risks of missing a secondary hypothalamic or pituitary cause. Our case illustrates common pitfalls in the diagnosis and management of hypogonadotropic hypogonadism as a first manifestation of panhypopituitarism associated with partial empty sella syndrome. Although initial hypopituitarism symptoms could be subtle and nonspecific and could involve only one hormonal axis, a thorough evaluation of the pituitary function may identify additional deficiencies such as a subclinical chronic adrenal insufficiency that may become manifest during situations of increased physiological stress (infection, surgery/invasive procedures) with potentially life-threatening consequences.
